# Reducing maternal and neonatal mortality through integrated and sustainability-focused programming in Zambia

**DOI:** 10.1371/journal.pgph.0001162

**Published:** 2022-12-14

**Authors:** Aniset Kamanga, Lupenshyo Ngosa, Oluwaseun Aladesanmi, Morrison Zulu, Elizabeth McCarthy, Kennedy Choba, James Nyirenda, Caren Chizuni, Angel Mwiche, Andrew Storey, Hilda Shakwelele, Margaret L. Prust

**Affiliations:** 1 Clinton Health Access Initiative, Inc., Lusaka, Zambia; 2 Clinton Health Access Initiative, Inc., Boston, MA, United States of America; 3 Zambia Ministry of Health, Lusaka, Northern Province, Zambia; 4 Central Office, Zambia Ministry of Health, Lusaka, Zambia; Harvard Medical School, UNITED STATES

## Abstract

Reducing maternal and neonatal mortality is a critical health priority within Zambia and globally. Although evidence-based clinical interventions can prevent a majority of these deaths, scalable and sustainable delivery of interventions across low-resource settings remains uneven, particularly across rural and marginalized communities. The Zambian Ministry of Health and the Clinton Health Access Initiative implemented an integrated sexual, reproductive, maternal, and newborn health (SRMNH) program in Northern Province aimed at dramatically reducing mortality over four years. Interventions were implemented between 2018 and 2021 across 141 government-owned health facilities covering all 12 districts of Northern Province, the poorest performing province nationwide and home to over 1.4 million people, around six pillars of an integrated health system. Data on institutional delivery and antenatal and postnatal care were collected through the national Health Management Information System (HMIS). A community-based system for capturing birth outcomes was established using existing government tools and community volunteers since HMIS did not include community-based mortality. Baseline and endline population-based mortality rates were compared for program-supported areas. From the earliest period of population-based mortality reporting in 2019 to program end in 2021, there were statistically significant decreases of 41%, 45%, and 43% in maternal, neonatal, and perinatal mortality rates respectively. Between 2017 to 2021, institutional maternal, neonatal, and perinatal mortality rates across entirety of Northern Province reduced by 12%, 40%, and 41%, respectively. Service readiness and coverage for SRMNH services improved dramatically, supporting increased numbers of patients. Significant mortality reductions were achieved over a relatively short period, reinforced through an emphasis on sustainability and strengthening existing government systems. These results were attained through a consciously cost-efficient approach backed by substantially lower levels of external investment relative to prior programs, allowing many of the interventions to be successfully adopted by government within public sector budgets.

## Introduction

Most maternal and neonatal deaths are preventable [1], yet women and infants in Sub-Saharan Africa continue to die at a disproportionate and alarming rate: there were an estimated 295,000 maternal deaths and 2.5 million neonatal deaths globally in 2017 and 2.0 million stillbirths in 2019, with Sub-Saharan Africa accounting for 66%, 41%, and 42% of those deaths, respectively [2–4]. Although known clinical interventions can prevent most of these deaths, delivery of these interventions in a scalable and sustainable way continues to be a challenge across low- and middle-income countries [5].

Relative to other countries with available mortality data, Zambia ranks 141 out of 185 for maternal mortality and 162 out of 195 for neonatal mortality [6]. Women in Zambia face a lifetime risk of 1 in 100 of dying in pregnancy or childbirth, and 1 in 37 infants die in their first month of life [7]. However, significant improvements in maternal and infant outcomes have been made in recent decades. Zambia’s maternal mortality ratio decreased by more than half between 2000 and 2017 (from 528 to 213 maternal deaths per 100,000 live births) [2] and the neonatal mortality rate decreased from 37 to 27 neonatal deaths per 1,000 live births between 2001 and 2018 [7].

A number of strategic, policy and implementation-related decisions in Zambia contributed to this success between approximately 2000 and 2017. Nationally, the availability of health facilities with skilled birth attendants and outreach to communities to encourage facility delivery have been important drivers of mortality reductions. In 2018, 84% of deliveries occurred in health facilities and 80% of births were attended by a skilled provider [7], compared to 2001 when only 44% of deliveries occurred in health facilities and 43% of births were attended by a skilled provider [8]. However, compared to these national statistics only 72% of deliveries occurred in health facilities and 70% of births were attended by a skilled provider in Northern Province [7]. There have also been more targeted interventions that have attempted to demonstrate success in certain areas. Among the programs that contributed to this success include: Saving Mothers Giving Life (SMGL), a U.S. government funded district systems strengthening program implemented in four districts of Zambia (not including Northern Province) between 2013 and 2018; Zambia Integrated Systems Strengthening Program (ZISSP), a USAID-funded project that ran from 2010 to 2014 and focused on health worker training, community health systems, and data quality; Safe Motherhood 360+, a USAID-funded project in five provinces (not including Northern Province) from 2016 to 2020; and the Zambia Health Services Improvement Project (ZHSIP), a World Bank-funded results-based financing program implemented in parts of Northern Province from 2014 to 2019 aiming to increase the use and quality of maternal and child health services. However, despite these interventions implemented in various parts of the country, Northern Province remained largely without any major investment with its key maternal and child health indicators being among the poorly performing across the country.

Despite this earlier success, reducing maternal, neonatal and perinatal mortality still remained an urgent health sector priority within Zambia as of 2018. Maternal deaths in Zambia, as around the globe, are mainly attributed to infection, hemorrhage, hypertensive disorders of pregnancy, and complications of unsafe abortion, while neonatal mortality is mainly linked to intrapartum-related events, preterm birth complications, sepsis, congenital abnormalities, and pneumonia [9]. There are well-known clinical interventions to address the major drivers of maternal and neonatal mortality and stillbirths, including the interventions recommended in the EmOC package, but gaps remain in providing those services at the community level and linking women with the health system [10, 11]. Prior to the launch of the program, the gaps in community-level service provision and linkages to the health system were prominent in Northern Province.

### Intervention

In 2018, the Ministry of Health (MOH) of Zambia and the Clinton Health Access Initiative, Inc. (CHAI) partnered to implement an integrated sexual, reproductive, maternal, and newborn health (SRMNH) program that aimed to reduce maternal, neonatal and perinatal mortality by 40%, 40%, and 20%, respectively, over four years in the Northern Province of Zambia. Understanding that most preventable maternal deaths occur in the 24- to 48-hour window around childbirth, the strategy in Zambia was to establish the systems to identify complications early to prevent them becoming life-threatening, intervene as needed with simple, evidence-based interventions to ensure survival, and refer cases quickly to the appropriate health system level for proper treatment. The approach was underpinned by a focus on increasing access to modern contraceptives to allow women to plan their pregnancies.

The program was designed around six core elements or pillars of an integrated and functioning health system that must be in place to drive significant reductions in unplanned pregnancies, maternal and neonatal mortality and stillbirths: 1) information for women and adolescent girls about their reproductive health and rights to allow them make informed choices and control over their reproductive decisions; 2) clear protocols that describe the interventions, skills, commodities and equipment required at each level of the health system to guide the health care workers; 3) sufficient numbers of trained and mentored health care workers to deliver key interventions; 4) functional procurement and supply chain systems to ensure the reliable supply of affordable commodities, consumables and equipment necessary; 5) functioning and sustainable emergency transport, communication and referral systems to provide a lifesaving link between the community and each level of care, reinforced by detailed referral protocols and directories; and 6) robust management and information systems in place to monitor and sustain the integrated system. The pillars were used to design program activities across health service types and levels of the health system across Northern Province (**Table 1**). This program embodied a ‘Network of Care’ approach that prioritizes linkages across all levels of care to ensure client-centered, effective, efficient operations and collaborative learning [12].

**Table 1:** Key intervention objectives and activities by health system level.

Health system level	Objectives	Key activities implemented by CHAI and MOH
**Community level**	Strengthen processes to effectively track pregnant women and newborns and refer them to primary health facility to receive appropriate care, particularly during childbirth and in the 48 hours after childbirth.	• Engaged community senior and sub-chiefs in the province to gain support and influence on SRMNH programs • Trained 1,450 SMAGs to track pregnant mothers and their newborns, identify complications, refer to nearest health facility, and report outcomes • Deployed motorbike ambulances (MBAs) and trained and equipped volunteer MBA riders to provide transport from communities to facilities for SRMNH and other services, and established sustainable systems for MBAs to be owned and maintained by communities • Trained 121 CHAs and equipped them with motorcycles to conduct community outreach and client tracking • Trained and supported 360 peer educators to reach and refer adolescents at community level and within schools • Trained 759 community-based distributors (CBDs) to provide family planning services at community level and conduct referrals where necessary • Hosted weekly radio programs on local stations to share information on maternal and neonatal danger signs
**Facility level**	Build capacity of health workers to identify, manage and treat emergency complications and perform routine obstetric and newborn care and ensure that health facilities are equipped with the medicines, equipment and supplies by strengthening pharmacy inventory management and ordering processes.	• Provided training to over 370 health workers on SRMNH services and ensured that all 85 EmOC-level facilities had health workers with EmOC training and mentorship • Trained 148 health care workers trained to become mentors to provide mentorship sessions to other healthcare workers. • Supported quarterly mentorship to all program-supported facilities using a mentorship model that not only focused on clinical skills but also systems troubleshooting • Conducted catalytic procurement of medicines and commodities in the beginning of the program to ensure that trained staff would have supplies to begin providing services • Established district-level teams of clinical and pharmacy staff to devise local solutions that address local bottlenecks in the supply chain • Provided training, tools, and ongoing guidance to strengthen Maternal and Perinatal Death Surveillance and Response (MPDSR) meetings and actions • Introduced and monitored use of the Non-pneumatic Anti-Shock Garment (NASG) at the primary care level to facilitate stabilization and transfer of women with obstetric hemorrhage
**District/ Provincial**	Strengthen capacity of district and provincial managers to provide oversight and accountability at all levels and take decisions based on data.	• Trained provincial and district health managers to provide oversight and supervision for program interventions–training, mentorship, supplies and equipment, referrals, and information systems • Conducted routine program review meetings to review progress, identify gaps and take corrective measures to improve performance • Developed dashboard for real time monitoring of the data which led to rapid programmatic adjustments
**Health system linkages**	Enhance communication and referral systems and improve access to emergency transport	• Mapped referral pathways from community to facility levels, designed and introduced clinical referral protocols, communication protocols and directories, a maternal referral form, and patient handover guidelines • Optimized district ambulance response system to improve efficiency during obstetric emergencies • Deployed motorbike ambulances (MBAs) for emergency transport to transport mothers and babies from communities to the nearest level of health care • Facilitated feedback mechanisms between the different levels of the health system, enabling sharing of learnings

The program in Zambia was inspired by lessons and results from Northern Nigeria, where a set of interventions produced impressive reductions in maternal mortality (37%), neonatal mortality (43%), and perinatal mortality (27%) over five quarters of program implementation across three states and covering a population of approximately 10 million people [13]. In the case of Nigeria, the success of the program was largely due to the implementation of a package of community-and facility-based interventions that had individually been shown to be effective in research settings [13, 14]. This program used a ‘Networks of Care’ approach [15] to create and reinforce parallel and horizontal linkages across all levels of the health system, including implementing community-based activities through local stakeholders and referral networks, and supporting facility-based activities such as training and mentoring, ensuring essential commodity and equipment availability, and enhancing health information management. After CHAI successfully transitioned out of the program, these results were sustained under the leadership of State and Federal Governments with key components subsequently adopted in other parts of Nigeria and this work was recognized with the ‘The Horizon Prize for the Birth Day’ for demonstrating a novel, safe and scalable solution for reducing maternal and perinatal mortality [16].

The program in Zambia aimed to achieve similar results by packaging community and facility-based interventions that addressed the country’s gaps in service delivery. Particular consideration was given to monitoring the community level, recognizing that consensus does not exist globally as to how best to establish a surveillance system that can be used to identify, review, and prevent maternal and neonatal deaths [17, 18]. Evidence suggests that death reviews are most useful when implemented as part of an overall package of support including training and mentorship [19]. Following the lead of CHAI’s Nigerian program, where the use of community mortality surveillance linked with death audits and reviews with the district health managers and facility staff was recognized as an important contributing factor to the overall success, a maternal and newborn maternal death surveillance system was included as a core component of the four-year program in Zambia. The data was both used for program improvement and to measure the change in mortality over time.

## Methods

A pre-post design was used to assess the impact of the SRMNH program on maternal, neonatal, and perinatal mortality rates in Northern Province, Zambia.

### Setting

This program was implemented from 2018 to 2021 in Northern Province of Zambia. Northern Province was selected by the government as the focal area for this work because it was one of the most rural and underperforming areas of the country on SRMNH-related outcomes and there were no other maternal and newborn health programs active in the province. The program was implemented in 141 government-owned health facilities across all 12 districts of Northern Province, encompassing 83% of all facility deliveries occurring in the province in 2018. Facility selection was undertaken using a multi-criteria decision analysis weighted sum model (WSM) [20, 21], prioritizing sites with high maternal mortality ratio, high neonatal mortality rate, high population headcount, high number of antenatal visits, and availability of a community health assistant (CHA). Although not all facilities were directly supported, work to strengthen the provincial and district systems reached all sites and included most of the province’s hospitals in the province, resulting in all women in the province being at least indirectly served by a program site. **Table 2** provides more detail on the program setting.

**Table 2:** Baseline characteristics of program setting^a^.

Characteristic	Value	
Area (sq. km)	77,650	
Population [23]	1,430,543	
Percentage of population in rural areas [23]	80.8%	
Number of women of reproductive age (15–49 years) [23]	316,172	
Number of expected births [23]	63,693	
Percentage of deliveries in health facilities [7]	72.2%	
Number of health facilities, by type (2018)	*Program-supported*	*Not program-supported*
Health posts	55	19
Health centers	79	30
District hospitals	5	1 ^b^
Regional hospitals	2	0

Information is shown for the whole area of Northern Province as of 2018.

Chilubi Island District Hospital was the only district hospital not included in the program. This site was not included due to being a mission hospital that did not provide some of the supported reproductive health services.

The COVID-19 pandemic spread across the globe during the course of program implementation. At the time that the pandemic began, most program trainings and initial mentorship activities were completed, and coordination relationships were established. Recognizing early that potential harm of disruption of essential services during such a crisis [22], the program worked both proactively and reactively to manage and respond to emergent issues and sustain routine care during this time. During this period Zambia imposed several periods of lockdown when MOH and CHAI staff were unable to travel or hold meetings, but facility staff and community volunteers continued carrying out their roles locally with virtual support and data reporting. While this constituted a change of plans, the context reinforced leadership at local levels and emphasized the advantages of a decentralized program management and operations team mostly located in Northern Province rather than the capital.

### Data sources

Data is presented from several sources. Routine facility-based data reported by facilities through the pre-existing, national health management information system (HMIS) was used to capture institutional mortality as well as other institutional service delivery indicators, as described below. In the absence of routine reporting of all deaths occurring within and outside health facilities, the program supported the government to establish community pregnancy and birth surveillance based on key informant reporting to produce population-based mortality estimates. This effort was in line with pre-existing government and community priorities and was seen as a test case for improved community mortality surveillance nationwide. To provide insight into how mortality gains were achieved, this paper includes data on resource availability from health facility assessments. These data sources are described below in further detail.

#### Routine facility-based data

On a monthly basis, all public sector health facilities in Zambia report to HMIS on aggregate disease burden and service delivery volumes. Data are entered by facility or district staff according to standardized forms and made available through the District Health Information System (DHIS2) platform. During this program, HMIS data were monitored by MOH and CHAI staff using dashboards in DHIS2 and in GoogleSheets. Data monitored by the program included the number of key SRMNH services provided (such as for contraceptives, antenatal care, post-natal care, and facility deliveries) (S1 Data) as well as health outcomes such as institutional maternal and neonatal mortality, and stillbirths (S2 Data).

#### Community pregnancy and birth surveillance data

While the HMIS system captures institutional mortality, there was limited data about births and deaths that occurred outside of health facilities, a problem recognized by the MOH leading to the approval in 2016 of pregnancy and birth tracking forms and a training manual for Safe Motherhood Action Groups (SMAGs). SMAGs are community volunteers focused on reproductive health, and the forms were designed to help SMAGs track basic information about women as they followed them throughout pregnancy and childbirth. This program built on the foundations of an ongoing program to both strengthen SMAG training and create a process for monthly aggregation and reporting of data on pregnancy and birth outcomes. As SMAGs were integral to both community surveillance and other programming, the community surveillance work was rolled out simultaneously with other program activities. In late 2018 and early 2019, the program provided training for facility in-charges, Community Health Assistants (CHAs), and 1,585 SMAG members from the 134 health centers and health posts supported by the program. Only the health centers and health posts with well-defined catchment populations reported community surveillance data, with hospitals serving as referrals sites (S3 Data).

The community surveillance data is hosted on the DHIS2 platform also used by MOH for HMIS data, helping to ensure data accessibility and sustainability. To encourage the use of data to drive decision-making, the program developed standardized dashboards and scorecards and conducted trainings on data use for Districts Health Directors, District Health Information Officers, and Maternal and Child Health Coordinators in 2019. The program continues to provide supportive supervision to SMAGs and their supervisors, conduct data quality checks, and host data review meetings with district and provincial staff. Data quality is checked by identifying outlier data points and making comparisons to other available data such as facility-based deaths in HMIS or deaths recorded in Maternal and Perinatal Death Surveillance and Response (MPDSR) data. Because of differences in inclusion criteria and process, these data sources were not expected to match exactly, but data were used to triangulate and provide basic checks.

Although the electronic system to capture community surveillance data was operational as of September 2018, reporting levels were limited in the early introductory period during scale up. Among the 134 program-supported health centers and health posts, 100% of facilities reported by March 2019. From that point until at least the end of 2021, monthly reporting rates remained at 98% or higher. The catchment area of each facility was broken into approximately 5 to 15 zones (zones designation pre-dated this program), and facility reporting represented an aggregation of data from available zones. Therefore, even when a facility reported in a given month, reporting coverage may vary based on how many zones were represented. As of April 2019, 91% (1014) of the total 1114 zones reported, and zonal reporting rates remained at 92% or higher through the endline period. Since mortality rates should be measured over a 12-month period for reliability, we consider the baseline period of reporting to be April 2019 to March 2020. This period was then compared to the latest 12-month period in the program, October 2020 to September 2021.

#### Health facility assessments

CHAI and MOH provincial and district staff conducted quarterly visits to all program-supported facilities to assess readiness to provide SRMNH services, generating data on service readiness gaps to guide program interventions (S4 Data). Data captured included the availability of skilled staff, equipment, and commodities to provide SRMNH services. Data was also captured on history of service provision for some items that are not tracked in HMIS. Assessments were conducted in person beginning in mid-2018 through 2019, and then through a combination of virtual and in-person visits in 2020 due to COVID-related restrictions. The final assessment was conducted in December 2020 with 2021 planned as a transition year with lighter program intervention. Data was collected on tablets in SurveyCTO using a form that was adapted from the WHO Service Availability and Readiness Assessment (SARA) and the Zambia Service Quality Assessment (SQA) tool. Data was available in real-time through a GoogleSheets dashboard, which allowed key decision makers in the program areas to interpret and use data.

### Data analysis

Indicators with binary outcomes were analyzed using a McNemar’s chi-squared test with matching by facility. Indicators with continuous data were analyzed using Wilcoxon signed-rank tests with matching by facility. Mortality data were analyzed using mortality rates as defined in **Table 3**. Mortality ratios presented are based on complete enumeration of all deaths identified in program-supported areas, and therefore, data is not subject to sampling error but may be affected by random variation and changes in case detection. In comparing the periods, a z-statistic was used to calculate the p-value of the difference between the mortality rates at different time points and model confidence intervals with the assumption that deaths and births are distributed according to a Poisson distribution [24]. The same z statistics were used to measure change in the service provision data. All analyses were performed in Excel and Stata 15.

**Table 3:** Definitions of mortality outcomes measured.

Outcome	Definition
**Population maternal mortality ratio (MMR)**	Maternal deaths occurring within or outside of health facilities of women who reside in a program area / Total number of live births born to women who reside in a program area * 100,000
**Institutional MMR**	Maternal deaths occurring in program supported facilities / Live births occurring in program supported facilities * 100,000
**Population perinatal mortality rate (PMR)**	All fresh stillbirths, macerated stillbirths, and early neonatal death (0–7 days) occurring within or outside of health facilities among infants born to women who reside in a program area / Total number of live births, fresh stillbirths, and macerated stillbirths born to women who reside in a program area * 1,000
**Institutional PMR**	All fresh stillbirths, macerated stillbirths, and early neonatal death (0–7 days) occurring in program supported facilities / Total number of live births, fresh stillbirths, and macerated stillbirths occurring in program supported facilities * 1,000
**Population neonatal mortality ratio (NMR)**	All neonatal deaths during the first 28 days of life (0–27 days) occurring within or outside of health facilities among infants born to women who reside in a program area / Total number of live births born to women who reside in a program area * 1,000
**Institutional NMR**	All neonatal deaths during the first 28 days of life (0–27 days) occurring in program supported facilities / Total number of live births occurring in program supported facilities * 1,000

### Ethical approvals

The community surveillance and facility assessment components were approved by the Zambian ERES Converge ethics review board in two separate protocols (reference numbers 2019-Jul-016 and 2018-Jul-017). Further research clearance was also obtained from the National Health Research Authority (NHRA) as well as the relevant authorities from Ministry of Health officials at national and sub-national level, including the facilities where data collection took place, and reinforced through review by CHAI’s internal Scientific and Ethical Review Committee.

## Results

### Mortality trends

Population maternal, neonatal, and perinatal mortality rates significantly declined in the catchment areas of the program facilities in Northern Province from baseline to endline according to community mortality surveillance data. These population rates are inclusive of community and institutional births and deaths using data from the community mortality surveillance system. From the first 12-month period of reporting (April 2019 to March 2020, following program launch in 2018) to the final 12-month period (October 2020 to September 2021) (**Table 4**), we observed a 41% reduction in population maternal mortality (MMR 181 to 106), a 45% reduction in population neonatal mortality (NMR 9.0 to 4.9), and a 43% reduction in population perinatal mortality (PMR 16.3 to 9.3).

**Table 4:** Population and institutional mortality rates.

**Population mortality data** ^**a**^	**Period 1: April 2019 to March 2020**	**Period 2: October 2020 to September 2021**		**Percent change from Period 1 to Period 2**	**P value** ^**b**^
No. of maternal deaths	60	32		n/a	n/a
No. of neonatal deaths	297	148		n/a	n/a
No. of perinatal deaths	545	281		n/a	n/a
No. of live births	33,160	30,051		n/a	n/a
No. of total births	33,459	30,204		n/a	n/a
Population MMR	181	106		-41.1%	0.013
Population NMR	9.0	4.9		-45.0%	< 0.001
Population PMR	16.3	9.3		-42.9%	< 0.001
**Institutional mortality data** ^**c**^	**Jan-Dec 2017**	**Jan-Dec 2019**	**Jan-Dec 2021**	**Percent change from 2017 to 2021**	**P value** ^**d**^
No. of institutional maternal deaths	54	70	67	n/a	n/a
No. of institutional early neonatal deaths ^c^	350	393	297	n/a	n/a
No. of institutional perinatal deaths	1,292	1,150	1,061	n/a	n/a
No. of institutional live births	38,151	60,723	53,829	n/a	n/a
No. of institutional total births	39,093	61,480	54,593	n/a	n/a
Institutional MMR	142	115	124	-12.1%	0.487
Institutional early NMR	9.2	6.5	5.5	-39.9%	<0.001
Institutional PMR	33.0	18.7	19.4	-41.2%	<0.001

a. Population mortality data comes from the community mortality surveillance system established by the government with program support.

b. P values are calculated using a z statistic.

c. Institutional mortality data comes from routine Health Management Information System (HMIS) reporting. Institutional mortality is shown for all public sector sites in Northern Province, including those support and not supported directly by the program.

d. In 2021, the government rolled out new HMIS reporting forms. In particular, the data collected around neonatal deaths changed substantially, and there were challenges due to COVID-19 with properly training staff to report on the new forms. This situation resulted in no data being reported on neonatal deaths in DHIS2 for 2021 as of the end of the year. The government plans to retrospectively enter early neonatal deaths into DHIS2 based on records kept through the MPDSR system, which was strengthened during the program based on improvements to community death reporting. For this analysis, we used district-level MPDSR data for early neonatal deaths. Late neonatal deaths and facility-level data were not available.

Institutional early neonatal mortality declined by 40% (NMR 9.2 to 5.5) and institutional perinatal mortality declined by 41% (PMR 33.0 to 19.4) between 2017 and 2021, according to HMIS data for all facilities in Northern Province (**Table 4**). Institutional maternal mortality reduced by 12% (MMR 142 to 124) in the same 4-year period. As illustrated in Table 4, the most dramatic reductions in institutional mortality rates were achieved from 2017 to 2019 (the program midpoint); these mortality rates were sustained through 2021.

### Health facility service readiness

Reductions in mortality were supported and enabled by dramatic improvements in health facility service readiness over the course of the program (**Table 5**), as measured by the health facility assessments. At baseline, none of the program-supported facilities had all the supplies and skilled staff necessary to provide basic emergency obstetric and newborn care (BEmOC), medical or surgical abortion, or post-abortion care services. By the endline, 92.9%, 90.0% and 71.8% were prepared to provide these services, respectively. Among the items required for service readiness bundles, it proved to be most challenging to consistently ensure availability of equipment, such as exam lights and tenaculum (results by individual item not shown).

**Table 5:** Facility characteristics at baseline, midline, and endline.

Characteristic	Baseline (July 2018) ^a^	Midline (December 2019) ^a^	Endline (December 2020) ^a^	Percent change from baseline to endline^b^	P value^b^
**Availability of supply and skill staff service bundles**					
Percentage of facilities with supplies and skilled staff available to provide BEmOC services ^c^	0	43.5	92.9	n/a	<0.001
Percentage of facilities with supplies and skilled staff available to provide medical or surgical abortion ^d^	0	60.0	90.0	n/a	0.003
Percentage of facilities with supplies and skilled staff available to provide post-abortion care ^e^	0	44.7	71.8	n/a	<0.001
**Availability of key individual commodities**					
Percentage of facilities with no stockout in the last 90 days: misoprostol	1.2	60.0	67.1	+5492%	<0.001
Percentage of facilities with no stockout in the last 90 days: magnesium sulphate	51.8	49.4	84.7	+63.5%	<0.001
Percentage of facilities with at least one long-acting reversible family planning method available in stock (implant or IUD)	87.1	76.5	63.5	-27.1%	0.858
Percentage of facilities with basic newborn care supplies available ^f^	48.2	95.3	100.0	+107.5%	<0.001
**Provision of BEmOC signal functions in last 90 days**					
Percentage of facilities administering parenteral antibiotics	65.9	80.0	97.7	+48.3%	<0.001
Percentage of facilities administering uterotonic drugs to treat post-partum hemorrhage ^g^	89.4	70.6	74.1	-17.1%	0.020
Percentage of facilities providing parenteral anticonvulsants for the management of pre-eclampsia/eclampsia	20.0	29.4	43.5	+117.5%	0.002
Percentage of facilities providing manual removal of placenta	40.0	34.12	49.4	+23.5%	0.258
Percentage of facilities providing removal of retained products of conception	44.7	44.7	84.7	+89.5%	<0.001
Percentage of facilities performing assisted vaginal delivery	24.7	48.2	37.7	+52.6%	0.028
Percentage of facilities performing basic neonatal resuscitation	81.2	89.4	82.4	+1.5%	0.827

a. Data in this table comes from program health facility assessments. Values represent the percentage of all facilities at the health center level or higher that met criteria (n = 85).

b. Percent change is “n/a” or not available where the baseline value was 0. Significance testing is based on a McNemar’s chi-squared test using matched data by facility.

c. BEmOC service readiness bundle included: Sterile gloves, fetoscope, blood pressure apparatus, functional thermometer, partograph manual vacuum aspiration kit, examination light, sterile scissors/blade, cord tie, mucous extractor, bag and mask, at least one broad-spectrum antibiotic, uterotonics, anticonvulsants, antihypertensives, and staff trained and mentored in BEmOC.

d. Service readiness for medical and surgical abortion was measured only in zonal health centers and hospitals according to national policy on eligibility to provide this service (n = 10). Medical abortion service readiness bundle included: misoprostol, mifepristone, and trained and mentored staff. Surgical abortion service bundle included: manual vacuum aspiration kit, speculum, tenaculum, trained and mentored staff trained and mentored in medical and surgical abortion.

e. Post-abortion care service readiness bundle included: manual vacuum aspiration kit, speculum, tenaculum, ringed forceps, at least one broad-spectrum antibiotic, misoprostol, oxytocin, and staff trained and mentored in post-abortion care.

f. Newborn care package included: sterile scissors/blade, cord tie, mucous extractor, and bag and mask.

g. Note that whereas the WHO signal function “administer uterotonic drugs” does not differentiate between prevention and treatment, the data collected in this program is specifically related to treatment of post-partum hemorrhage.

Reductions in stockouts were observed for key commodities tracked. For misoprostol, 1.2% of facilities reported no stockout of misoprostol in the last 90 days at baseline, and by endline 67.1% of facilities had no stockout in the last 90 days. Magnesium sulphate was more available at baseline, with 51.8% of facilities having no stockout, but the availability improved such that 84.7% of facilities had no stockout at endline. Although the availability of most commodities improved steadily over the grant period, the percentage of facilities with either an IUD or implant in stock reduced over the program period from 87.1% to 63.5% due to global supplier shortages and reduced funding for family planning commodities by donors.

Because the provision of BEmOC signal functions is not reliably tracked in HMIS, we assessed this through facility assessments. Significant increases were observed from baseline to endline in the percentage of facilities providing parenteral antibiotics (from 65.9% to 97.7%), parenteral anticonvulsants for the management of pre-eclampsia/eclampsia (from 20.0% to 43.5%), removal of retained products of conception (from 44.7% to 84.7%) and assisted vaginal delivery (from 24.7% to 37.7%) in the last 90 days. The percentage of facilities performing basic neonatal resuscitation was high at baseline and remained high; while the provision of uterotonic drugs to treat post-partum hemorrhage was also high at baseline, the percentage of facilities providing this service decreased over the program (from 89.4% to 74.1%). Routine use of uterotonics to prevent post-partum hemorrhage was not captured but may have contributed to fewer cases of post-partum hemorrhage requiring treatment. Provision of manual removal of the placenta in the last 90 days was 40.0% at baseline and increased slightly endline (49.2%).

### Facility-based routine data

According to data from national HMIS reporting, the volume of key SRMNH services provided at program facilities increased dramatically from the year before the program began (2017) to the final year of the program (2021) (**Table 6**), particularly the provision of contraceptives, as measured by couple-years of protection (88.7% increase). While the number of first ANC visits reported was relatively stable, the percentage of first ANC visits that occurred in the first trimester nearly doubled from 22.5% to 42.7%. There was a more modest increase across Northern Province in the percentage of estimated deliveries that happened in health facilities (from 63.4% to 78.6%) and in the percentage of estimated live births where postnatal care was received at two to six days (from 43.0% to 46.4%). These indicators of facility deliveries and post-natal care rose to a peak in 2019, and then fell slightly by 2021.

**Table 6:** Service delivery volumes and coverage^a^.

	Jan-Dec 2017	Jan-Dec 2019	Jan-Dec 2021	Percent change from 2017 to 2021	P value ^b^
**Volume of key services**					
Contraceptive years of protection provided for modern contraceptive methods (pills, injectables, implants and IUDs)	54,947	117,234	81,026	+88.7%	<0.001
First antenatal care visits	49,014	59,783	50,565	-1.0%	0.407
Institutional deliveries	34,385	48,962	41,044	+17.6%	0.003
Postnatal care visits	21,502	36,278	21,989	+19.1%	0.654
**Coverage of key services**					
Percentage of first antenatal care visits that took place before 14 weeks	22.5	63.2	42.7	+89.8%	<0.001
Percentage of estimated deliveries occurring in facilities ^c^	63.4	92.6	78.6	+23.8%	<0.001
Percentage of estimated live births where postnatal care is received in 2–6 days ^c^	43.0	75.0	46.4	+7.9%	<0.001
Percentage of facilities providing LARCs in the last 90 days (at least one implant and one IUD)^d^	8.2	36.5	31.8	+288%	<0.001

a. Data in this table comes from national HMIS reporting. Values shown reflect data reported from all program-supported facilities (n = 141) unless otherwise noted.

b. P values for continuous variables represent a comparison of baseline and endline using a Wilcoxon signed rank test with matching by facility. The p value for the binary outcome variable (LARC provision) represents a comparison of baseline and endline using a McNemar’s chi-squared test with matching by facility. P values for other service coverage portions are based on a z statistic for the value for all facilities combined.

c. Values reflect all of Northern Province. Deliveries and live births are estimated for the whole province by the Central Statistics Office, so the numerators used (facility deliveries and PNC visits) are from all facilities in the province.

d. For indicators referring to the last 90 days, data is shown for October to December of each year. Values reflect the percentage of facilities eligible to provide LARCs (n = 85).

## Discussion

This SRMNH program introduced an integrated, systems-focused approach towards enhancing SRMNH services and outcomes in Zambia’s Northern Province over a period of four years. From 2019 to 2021, the population maternal, neonatal, and perinatal mortality reduced by 41%, 45%, and 43%, respectively, and from 2017 to 2021 the institutional maternal, early neonatal, and perinatal mortality reduced by 12%, 40%, and 41%, respectively. These dramatic mortality reductions are on par with reductions seen over considerably longer periods in Sub-Saharan Africa. For example, there was a 38.2% reduction in maternal mortality in the period from 2000 to 2017 [2] and the 40.0% reduction in neonatal mortality in the period from 1990 to 2017 [3].

A similarly rapid reduction of 41% in population maternal mortality and 26% in facility perinatal mortality was reported previously by the Saving Mothers, Giving Life (SMGL) program in four districts in Zambia [25]. These results were achieved with the investment of $200 million over five years in Zambia and Uganda [26]. By contrast this program was designed with a focus on sustainability and scale from the outset. By operating directly through government systems, expanding and strengthening activities already largely in place, and ensuring a genuine partnership between the MOH and CHAI, the program was able to maintain a cost-efficient approach that remained affordable for the Zambian Government following program transition. As early as the second year (2019) of this four-year program, the program stakeholders began to engage policy makers at national, provincial and district levels in conversations to describe program interventions, results, and costs. The program developed a costing tool that allowed policy makers to explore various scenarios for scale-up and continuation of interventions. Following these engagements, government funds were allocated in provincial and district plans beginning in 2020 to sustain priority program interventions including health worker mentorship, strengthening of adolescent health activities, community engagement activities, data review processes for community mortality surveillance and MPDSR, community emergency transport using motorbike ambulances, and use of NASGs for obstetric hemorrhage. This means that going forward the government will fund and implement these priority activities without support from donors. While not all program activities will be sustained, the government was able to go through a process to determine which activities had the strongest supporting evidence and were affordable for continuation. While neonatal and perinatal mortality reductions were similar for population and institutional mortality, the population maternal mortality decreased more substantially than the institutional mortality. This finding may reflect that the program had an influence on ensuring that women were more able to reach facilities in the event of complications, due to stronger referral and transport systems, and were therefore less likely to die in the community, as reflected in population mortality declines. But some referrals still arrived too late for facilities to act, and this was particularly true in the context of the COVID-19 pandemic. Another important factor in the difference between population and institutional mortality is that the population mortality measures include women who resided in program supported areas whereas institutional mortality measures include women who died at a program-supported facility, regardless of their place of residence. Anecdotally and based on MPDSR data triangulation, it seems that there was a trend towards more women being referred from other provinces to facilities in Northern Province as services there improved. Even in the context of improved services in Northern Province, some referrals were made too late to prevent mortality. We believe that these factors together may have led to a more modest reduction in institutional mortality and serve to underline the importance of continued and expanded service improvements.

Health service availability increased during the program period, and the program realized corresponding improvements in service coverage. Service readiness at baseline was low and aligned with similar studies in the region [27, 28], though the results shown in this paper are particularly low even by regional standards since we used definitions of facility readiness that required facilities to have all items in the service bundles available. Despite the lack of one of more items at baseline, many facilities were providing BEmOC signal functions. The performance of BEmOC signal functions in the last 90 days at baseline in this study is slightly higher than a 2014–2015 assessment in public sector sites in Zambia [29], possibly suggesting that some improvements had occurred in years immediately prior to the baseline. The dramatic increase in the availability of supplies and trained staff during the program likely served not only to increase the percentage of facilities providing key services such as BEmOC signal functions but also to improve the quality of care, though quality of care was not directly measured. The volume of institutional deliveries and post-natal care visits increased, as did the percentage of estimated deliveries receiving these services, which can likely be attributed to the work of SMAGs to encourage pregnant women to seek these services and to improvements in quality of care such that women were more eager to seek out services.

The latter half of this four-year program was implemented during the global COVID-19 pandemic. Initial health worker and community volunteer trainings planned as a part of the program were largely held in-person prior to the outbreak of COVID-19. However, many planned activities, such as health worker mentorship and management and data use meetings were interrupted by lockdowns and diversion of health worker time to COVID-related activities. During the height of the pandemic the program partners worked to ensure the continued provision of SRMNH services. For example, mentorship was provided by senior health workers in-house and remotely using the mentorship framework created by the program and through tools like WhatsApp groups. SMAG visits to households continued under government guidelines and management meetings transitioned to remote platforms where possible. The program adapted to redirect resources towards providing personal protective equipment to community and facility workers and training on infection prevention and control to ensure SRMNH services continued in a safe manner. The impact of COVID-19 can be observed in service coverage indicators such as the percentage of first antenatal care visits that took place in the first trimester, estimated deliveries occurring in facilities, estimated live births where postnatal care is received in two to six days, and facilities providing LARCs in the last 90 days. For each of these indicators, service coverage increased dramatically from 2017 to 2019, and then fell again slightly by 2021. While 2021 values remain well above 2017 levels, local stakeholders indicated that lockdowns and resistance to attending health facilities in 2020 and 2021 impacted data trends. But the sustained gains in health outcomes during this unprecedented time speaks to the resilience of the health system. Similarly, institutional maternal and perinatal mortality was lowest in 2019 and rose slightly by 2021, also likely due to the impact of COVID-19 both directly and indirectly in causing women to deliver at home and be referred late with complications. In 2021 when COVID-19 testing became widely available in maternity wards in Northern Province, at least three maternal deaths were documented to be among women with confirmed COVID-19. These cases were included in maternal mortality calculations. In addition to the impact of the COVID-19 pandemic, it should also be noted that this program was implemented during a period of fiscal challenges experienced by the Zambian government which escalated into the country defaulting on the debt repayments. As a result, the government health budget was constrained, which makes it even more remarkable that districts were able to commit to continuation of key program-initiated activities through sustainability and transition plans.

### Strengths and limitations

Various sources of mortality data were carefully considered by the program [30]. National mortality data is available approximately every five to seven years from the Zambia Demographic and Health Survey (ZDHS), though this is not frequent enough for assessing program impact or informing real time programming, and data is not available at sub-national level. Household or other survey-based approaches are not cost-effective and have limited potential for integration into routine systems. Deaths that occur in public sector health facilities are tracked in the national HMIS, but this data source misses deaths that occur in private facilities, in transport between facilities, or in communities. In Zambia, there is also a reporting system associated with the MPDSR process, but this data source also misses many non-institutional deaths due to the lack of a system for information about these deaths to flow to facilities. Considering these gaps in mortality surveillance, the program opted to support ongoing Government ambitions to develop a community maternal and newborn mortality surveillance system and track this data in parallel with facility-based reporting from HMIS. The community surveillance system was established using existing government tools and community health volunteers. By 2021 and with the support and example of this work in Northern Province, the government added a requirement to track community-based maternal deaths in the HMIS system.

At the same time, this approach to mortality surveillance has several limitations. First, the community surveillance system was launched in parallel with the program, so the first year of population-based mortality data (April 2019 to March 2020) represents a period in which the program interventions were already underway and not a true baseline. Program activities were well underway by late 2018. For that reason, we suspect the change in mortality measured by that data is a conservative estimate of the true program impact. Second, this evaluation lacks a comparison to a counterfactual, non-program supported area. Northern Province was purposefully selected as the site for program implementation because of its poor health outcomes relative to other areas of Zambia. This means that the Northern Province context is not easily compared to other areas, particularly in light of other programs that were active in other parts of Zambia at the same time. Additionally, the community mortality surveillance data was only available in Northern Province and institutional mortality data in other areas of Zambia did not receive data quality reviews as in Northern Province. Third, institutional mortality data is prone to selection bias because it represents outcomes for individuals that sought and received care in program-supported facilities. Not all individuals receiving services at program-supported facilities resided in the immediate catchment area of the facility–large referral facilities may receive patients from non-supported sites and even smaller sites in this program received referrals from outside of Northern Province. The triangulation of population and institutional mortality data is an effort to address this limitation. Fourth, as the interventions that were part of this program were implemented simultaneously and in an integrated fashion, it is not possible to ascertain the relative impact of individual program components.

Despite these limitations, this evaluation approach has a number of strengths. Our analysis integrates data on availability of and access to key SRMNH services, which are essential to demonstrating ultimate impact on mortality. Two sources of mortality data are presented for triangulation and validation. While this pre-post evaluation approach without comparison group has some limitations, it has been considered justifiable for projects using best practice interventions that have been previously proven to work under similar conditions [11, 31]. Finally, the community surveillance approach used for population mortality data was established to be beneficial not only for purposes of program evaluation but as a sustainable solution for capturing community mortality data, strengthening the ability of the government to carry out MPDSR reviews for all community and facility deaths, and increasing levels of community buy-in and stakeholder support, which can in turn facilitate further reductions in mortality [17, 32]. While our approach cannot completely isolate the impact of this program from generalized ecological trends, there were no major partner-supported reproductive health programs taking place in Northern Province at the time of this work, which supports impact attribution for this program.

### Conclusions

Reducing maternal and newborn mortality is one of the most important global health goals, as demonstrated in the Sustainable Development Goals which aim to reduce the global MMR to less than 70 per 100,000 births, with no country having a maternal mortality rate of more than twice the global average by 2030. Although the major causes of death are known and effective clinical interventions exist to address those causes, too many women and babies are lost each day. This SRMNH program in Zambia’s Northern Province demonstrated that significant mortality reductions can be achieved in challenging settings through investments in existing government systems and structures. Prior to the start of program, Northern Province was one of the most underperforming regions in Zambia for maternal and newborn health and program successes were realized through the high-impact, integrated SRMNH interventions that simultaneously addressed multiple facets and potential bottlenecks to quality service delivery, including community awareness and mobilization, policies and protocols, health workforce knowledge and skills, availability of medicines and equipment, transportation and referral systems, and information and management systems. It is critical to address all of these areas in an integrated manner, rather than picking out a single weakest link in order to strengthen the full system. Further, in order to ensure that gains are sustained over time, interventions must place public sector systems and stakeholders at the center of the work rather than building parallel systems, while supporting the government to develop and implement sustainability plans. The gains observed in Northern Province are significant and represent scores of maternal and newborn deaths averted. But despite the impact of a reinforced integrated system that will support pregnant women and their newborns long after project completion, it is undeniable that far too many mothers and newborn babies will continue to die, each one representing a tragedy for the individual, their family and their wider community. The work to improve health services and outcomes in Northern Province remains incomplete, but this program can provide the foundation for further improvement and a valuable model for similar settings.

## Supporting information

S1 Data HMIS service delivery dataset. (XLSX)

S2 Data Institutional mortality dataset. (XLSX)

S3 Data Population mortality dataset. (XLSX)

S4 Data Facility assessment dataset. (XLSX)

S1 Text Inclusivity in global research. (DOCX)
