# Supplementary material for: Reducing maternal and neonatal mortality through integrated and sustainability-focused programming in Zambia
Source: PLOS Glob Public Health. 2022 Dec 14;2(12):e0001162. doi: 10.1371/journal.pgph.0001162 (PMC10021549; doi:10.1371/journal.pgph.0001162)
Supplement: S1 Text — (DOCX) [file pgph.0001162.s005.docx]

Inclusivity in global research

PLOS’ policy on inclusivity in global research aims to improve transparency in the reporting of research performed outside of researchers’ own country or community and ensures that PLOS publications reporting global research adhere to high standards for research ethics and authorship. Authors of relevant research articles may be asked to complete the questionnaire below, which outlines ethical, cultural, and scientific considerations specific to inclusivity in global research. This questionnaire may be requested when researchers have travelled to a different country to conduct research, if research uses samples collected in another country, research with Indigenous populations or their lands, or if research is on cultural artefacts. Researchers travelling to another country solely to use laboratory equipment will not normally be required to complete the questionnaire. However, the questionnaire can be requested at the journal’s discretion for any submission – if you have been requested to complete this questionnaire by the PLOS journal you submitted to, please do so.

Please complete the questionnaire below and include this as a Supporting Information file with your manuscript. Note that if your paper is accepted for publication, this checklist will be published with your article in the supporting information files. Please ensure that you reference the checklist in the main body of your manuscript. We suggest adding a subsection ‘Inclusivity in global research’ to your Methods section and adding the following sentence: “Additional information regarding the ethical, cultural, and scientific considerations specific to inclusivity in global research is included in the Supporting Information (SX Checklist)”

The questions have been designed to be applicable to a wide range of study types, and there are subsections for both human subjects research and non-human subjects research. If any of the questions are not relevant to your research please mark them as “N/A” as appropriate.

**Ethical considerations, permits and authorship**

*This section is applicable to all research types.*

Provide details as to who granted permissions and/or consent for the study to take place in the Methods section of your manuscript. This should include the names of **all** ethics boards, governmental organizations, community leaders or other bodies that provided approval for the study. If individuals provided approval refer to these people by their role or title but do not list their name(s).

**Reported on page number: 13**

**This work was approved by:**

- **Zambia ERES Converge ethics review board**
- **Zambian National Health Research Authority (NHRA)**
- **Relevant authorities from Ministry of Health officials at national and sub-national level, including the facilities where data collection took place**
- **CHAI’s internal Scientific and Ethical Review Committee**

If there were any deviations from the study protocol after approval was obtained please provide details of these changes in the Methods section of your manuscript.

**Reported on page number: N/A**

Did this study involve local collaborators that are residents of the country where the research was conducted or members of the community studied? If you do not have any authors from said communities, please provide an explanation for this below.

**This work involved local Zambia collaborators that are residents of Zambia where the research was conducted. The Zambian collaborators that met the ICMJE criteria for authorship have been included as authors, including: Aniset Kamanga, Lupenshyo Ngosa, Morrison Zulu,** **Kennedy Choba, James Nyirenda, Caren Chizuni, Angel Mwiche, and Hilda Shakwelele.**

Everyone listed as an author should meet PLOS’ criteria for authorship and all individuals who meet these criteria should be included in the author byline, rather than the acknowledgements. Authorship criteria is based on the International Committee of Medical Journal Editors (ICMJE) Uniform Requirements for Manuscripts Submitted to Biomedical Journals - for further information please see here: <https://journals.plos.org/plosone/s/authorship>.

**Human subjects research (e.g. health research, medical research, cross-cultural psychology)**

Did you obtain written informed consent from a representative of the local community or region before the research took place? How did you establish who speaks for the community? Details of written informed consent obtained from study participants should be reported separately in the Methods section of your manuscript.

**This research was determined to be non-human subjects research and thus individualized written informed consent was not required or obtained.**

**However, it was public health research with important implications for maternal and newborn health in rural Zambia. As such, the team requested verbal consent from government representatives at the district, provincial and central levels and verbal consent from traditional chiefs and Neighborhood Health Committees (NHC) to carry out the work and for their support in engaging the community in program interventions. NHCs are committees appointed by the community to engage with the health facility staff and weigh in on community health issues.**

How did members of the local community provide input on the aims of the research investigation, its methodology, and its anticipated outcome(s)?

**Government stakeholders at the provincial and central levels were engaged as investigators in the study protocol and reviewed and approved all study materials. Other stakeholders such as traditional chiefs and Neighborhood Health Committees, as members of the local community and appointed by the local community to engage with health facility staff, were consulted regarding the approach to measuring change in maternal, neonatal and perinatal mortality.**

When engaging with the local community, how did you ensure that the informed consent documents and other materials could be understood by local stakeholders?

**N/A – As this study was determined not to be human subjects research and did not enroll individuals, informed consent documents were not requested.**

Will the findings of the research be made available in an understandable format to stakeholders in the community where the study was conducted (e.g. via a presentation, summary report, copies of publications, etc.)? Please provide details of how this will be achieved.

**Throughout the study period, preliminary results were presented and reviewed in meetings with government stakeholders at the district, provincial and central levels. These results will be further presented at the forthcoming 10^th^ National Health Research Conference. This is a biennial local and International Research Conference which hosts dedicated discussions of the most recent scientific innovations, and discoveries, as well as practical challenges, encountered and solutions adopted for improving the health of the population. All provincial and district health officials attend this conference which helps to disseminate about new findings of research work. At the community level, the program from inception has engaged traditional leaders and gate keepers in sharing the objectives of the program. Local chiefs and village headmen were approached to work with the program and several program review meetings have been held with these community leaders to share the program successes and challenges. Recently, the program held a community meeting where all chiefs from the region were brought together to talk about maternal health issues in the province and shared the data attained by the research. In addition, each facility holds quarterly Health Centre Committee (HCC) meetings where healthcare workers, traditional leaders and communities come together, similar to town hall meetings where they discuss health outcomes and performance of the facilities. The HCC meeting is a vehicle for social participation and accountability in health systems. Results have already been shared in these meeting set-ups.**

**Non-human subjects research using specimens/ animals collected as part of the study, or those housed in archival collections. Examples include archaeology, paleontology, botany and zoology.**

Did the permission you obtained from a local authority to perform the study include an agreement on access to outputs and benefit sharing? This may include procedures to enable fair distribution of the benefits and resources arising from the research performed. Please include any details of Prior Informed Consent and Benefit Sharing Agreements obtained. These may be required by field-specific regulations, for example the Convention on Biological Diversity (CBD) and the associated Nagoya Protocol.

**N/A**

If the material used in your study was imported, please A) provide the year it was imported and B) indicate whether permits were obtained to import/export the materials used, C) provide details of any permits obtained. If this information is not available, please indicate this.

**N/A**

If you used archival specimens, please state how the material used in your study was acquired by the institute it is held in and provide details of any permits obtained for the original excavations/ sample collection. If this information is not available, please indicate this.

**N/A**

How was the potential cultural significance of the materials collected in your study to local communities considered in your research design? Were Indigenous peoples and/or local researchers and institutions involved with archaeological excavations / collection of specimens? If so, please provide a description of their involvement.

**N/A**

If your manuscript includes photographs of human remains please indicate whether authors obtained permission from descendants or affiliated cultural communities to do so.

**N/A**
